# What Makes a Successful Donor? Fecal Transplant from Anxious-Like Rats Does Not Prevent Spinal Cord Injury-Induced Dysbiosis

**DOI:** 10.3390/biology10040254

**Published:** 2021-03-24

**Authors:** Emma K. A. Schmidt, Pamela J. F. Raposo, Karen L. Madsen, Keith K. Fenrich, Gillian Kabarchuk, Karim Fouad

**Affiliations:** 1 Neuroscience and Mental Health Institute, University of Alberta, Edmonton, AB T6G 2R3, Canada; ekschmid@ualberta.ca (E.K.A.S.); fenrich@ualberta.ca (K.K.F.); kabarchu@ualberta.ca (G.K.); 2 Faculty of Rehabilitation Medicine, University of Alberta, Edmonton, AB T6G 2R3, Canada; praposo@ualberta.ca; 3 Department of Physical Therapy, University of Alberta, Edmonton, AB T6G 2R3, Canada; 4 Division of Gastroenterology, Faculty of Medicine and Dentistry, University of Alberta, Edmonton, AB T6G 2R3, Canada; karen.madsen@ualberta.ca

**Keywords:** spinal cord injury, fecal microbiota transplant, inflammation, anxiety, rehabilitation

## Abstract

**Simple Summary:**

Spinal cord injury disrupts the composition of gut bacteria and increases the prevalence of anxiety-like and depressive-like behaviours. We have previously shown that a fecal transplant from uninjured donor rats prevents both injury-induced microbiota changes and the development of anxiety-like behaviour. In the present study, we aimed to determine whether donor selection would influence the efficacy of a fecal transplant after spinal cord injury. We found that a fecal transplant from uninjured donor rats with increased anxiety-like behaviour was not only ineffective in preventing injury-induced microbiota changes, but it also increased intestinal permeability and anxiety-like behaviour of the recipient rats. The results of this study emphasize the importance of optimal donor selection for successful fecal transplant treatment following spinal cord injury.

**Abstract:**

Spinal cord injury (SCI) causes gut dysbiosis and an increased prevalence of depression and anxiety. Previous research showed a link between these two consequences of SCI by using a fecal transplant from healthy rats which prevented both SCI-induced microbiota changes and the subsequent development of anxiety-like behaviour. However, whether the physical and mental state of the donor are important factors in the efficacy of FMT therapy after SCI remains unknown. In the present study, rats received a fecal transplant following SCI from uninjured donors with increased baseline levels of anxiety-like behaviour and reduced proportion of *Lactobacillus* in their stool. This fecal transplant increased intestinal permeability, induced anxiety-like behaviour, and resulted in minor but long-term alterations in the inflammatory state of the recipients compared to vehicle controls. There was no significant effect of the fecal transplant on motor recovery in rehabilitative training, suggesting that anxiety-like behaviour did not affect the motivation to participate in rehabilitative therapy. The results of this study emphasize the importance of considering both the microbiota composition and the mental state of the donor for fecal transplants following spinal cord injury.

## 1. Introduction

Spinal cord injury (SCI) causes damage to the spinal cord and disrupts the physical and mental well-being of individuals with SCI [1]. In addition to motor and sensory deficits, SCI can cause impairments in autonomic, immune and bowels functions as well as disturb the composition of the gut microbiota (termed dysbiosis) [2,3,4,5]. In a rat model of SCI, we have previously prevented SCI-induced dysbiosis by transferring fecal matter from uninjured donor rats into recipient rats immediately after SCI. This fecal microbiota transplant (FMT) from uninjured, non-anxious-like rats not only successfully re-established a healthy microbiota composition after injury, but also improved symptoms of anxiety-like behaviour [2].

Clinically, FMT is defined as the administration of fecal matter solution from a healthy donor into the intestinal tract of a recipient [6,7]. The use of FMT in clinical trials has most commonly been to treat *Clostridium difficile* infections and irritable bowel disease, however case reports have demonstrated beneficial results of an FMT for individuals with Parkinson’s disease, multiple sclerosis, Tourette syndrome, autism and epilepsy [8,9,10,11,12,13,14]. Unfortunately, the definition of a healthy donor is less straightforward. Currently, donors are selected primarily to exclude known pathogens and mitigate the risk of transferring infectious diseases [15,16,17,18]. While ensuring recipient safety is a priority above all, research on optimal donor selection beyond the exclusion of transmissible pathogens is still at an early stage [15,19]. Although the choice of donor does not influence the efficacy of FMT to treat *Clostridium difficile* infections (currently the only FDA approved use of FMT [20]), it is unknown how critical donor selection is to treat diseases and disorders with more complex host-microbiota interaction, such as SCI [9,21].

In the present study, we aimed to determine whether the mental state of FMT donor rats would influence the therapeutic benefits of FMT after SCI. Rats who displayed naturally reduced baseline activity levels and increased anxiety-like behaviour (referred to as *anxious* donors) were selected as FMT donors. Notably these rats were uninjured and had similar alpha diversity to uninjured, non-anxious-like rats, which is important since a diverse microbial diversity has been shown to be an indicator of FMT success for treatment of ulcerative colitis and *Clostridium difficile* infections [19,22]. We therefore hypothesized that FMT from *anxious* rats would yield similar therapeutic benefits as FMT from non-*anxious* rats as in our previous research [2]. Here, rats in the experimental groups received either vehicle or FMT treatment for 3 days following a cervical contusion SCI and underwent 7 weeks of rehabilitative training in a reaching task targeting their impaired forearm. Fecal matter and plasma were collected throughout the experiment, and anxiety- and depressive-like behaviours were assessed at the end of the rehabilitation period. The inherently increased anxiety-like behaviour of the FMT donors was associated with a decreased abundance of *Lactobacillus* in their stool and thus in the FMT solution. Contrary to our hypothesis, FMT from *anxious* donors did not prevent SCI-induced gut dysbiosis and even resulted in some negative side effects. Rats which received the FMT displayed chronically increased anxiety-like behaviour, long-term alterations in local and systemic inflammation, and increased intestinal permeability. These results indicate that donor selection is critical for successful FMT following SCI and possibly other CNS injuries and diseases as well.

## 2. Materials and Methods

### 2.1. Animals

40 female adult Lewis rats (Charles River) were group housed (*n* = 5 per cage, experimental groups housed separately to avoid coprophagy) on a 12 h light-dark cycle and received ad libitum access to standard rat chow and water. During training periods, rats were food restricted to 10 g per rat per day (to encourage reaching for training pellets). Behavioural testing and all analyses were performed by an experimenter blinded to the experimental groups. Three groups of rats were used: SCI + vehicle (*n* = 15), SCI + FMT (*n* = 15), and FMT donors (*n* = 10). The two cages which displayed the highest baseline anxiety-like behaviour in the open field were chosen as uninjured age and sex matched fecal donors and were not trained in the single pellet grasping (SPG) training. SCI + vehicle and SCI + FMT groups were chosen to average each group’s pre-injury success rate in the SPG task. Data from previous experiments that were used to compare elevated plus maze behaviour and *Lactobacillus* levels were taken from genetically comparable rats from the same supplier with the same weight, handled the same amount, and received the same diet.

This study was approved by a local animal care and use committee (Health Sciences) at the University of Alberta and in accordance with the guidelines of the Canadian Council for Animal Care.

### 2.2. Experimental Timeline

**Figure 1:**
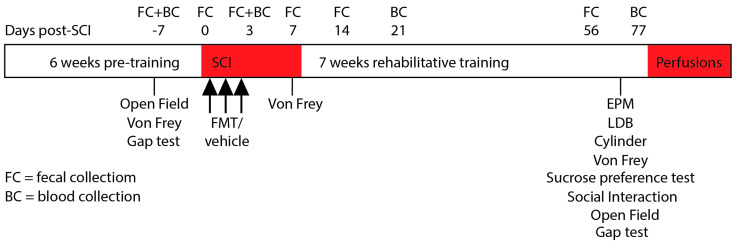
Experimental Timeline.

Prior to SCI, rats in the two experimental groups were pre-trained on the SPG task and underwent baseline testing on the open field, von frey and gap tests. The von frey test was also performed 7 days post SCI. Final behavioural testing was performed at the end of the rehabilitative training period between 63 and 77 days post injury. The FMT donors were handled daily to control for the potential effect of handling during rehabilitative training. Immediately following SCI and for two consecutive days thereafter, rats were gavaged with FMT or vehicle solution. Following 7 weeks of rehabilitative training on the SPG task, rats underwent behavioural testing. Fecal matter was collected for 16S rRNA analysis at baseline, on the day of injury (6–12 h after), 3, 7, 14 and 56 days post-SCI. Blood was collected to measure inflammatory plasma analytes at baseline, 3, 21 and 77 days post-SCI (Figure 1).

### 2.3. Single Pellet Grasping Training

The SPG protocols and equipment were used as previously described [23]. Rats were first acclimatized to the SPG double-window enclosure and each rat’s preferred paw was established by manually counting the number of left and right reaching attempts for a sucrose pellet. Once the preferred paw was established, the pellet dispenser was positioned so the rat could only reach the pellet with its preferred paw. Rats were trained to reach for a pellet on one side of the enclosure and then travel to the opposite end where another pellet was dispensed, etc. Training consisted of 10 min per rat per day, 5 days a week for 6 weeks prior to SCI. Rehabilitative training began 10 days following SCI and continued for 7 weeks. Training sessions were video recorded and analyzed offline. The total number of attempts made (rat reached towards the pellet) and number of successes (rat successfully reached, grasped and consumed the pellet) were quantified. Success rate was defined as the total number of successful attempts divided by the total number of attempts multiplied by 100. Once before SCI and again at the end of the rehabilitative period, rats were tested on a modified single pellet grasping gap test that prevents compensatory scooping strategies.

### 2.4. Spinal Cord Injury

SCI cervical contusions were performed as previously described [2]. Rats were anesthetized with isoflurane (5% induction; 2.5% maintenance; 50:50 air/oxygen mixture) and the dorsal neck was shaved and disinfected with 10% chlorhexidine digluconate (Sigma-Aldrich, St. Louis, MO, USA). The Infinite Horizons impactor (Precision Systems & Instrumentation) was used to deliver a 125 kdyn unilateral contusion 1.25 mm lateral to the midline (on the side of the preferred paw) at an angle of 15 degrees (towards midline) at cervical level 5. Synthetic braided sutures were used to suture the muscles and the skin was closed using 9 mm stainless steel clips. Buprenorphine was injected immediately after SCI and again 8 h after (0.03 mg/kg; subcutaneous; WDDC). Saline was injected (4 mL, subcutaneous) post operatively and bladders were manually expressed until voiding was re-established (within 2 days post SCI).

### 2.5. Behavioural Testing

#### 2.5.1. Light Dark Box

Rats were placed in the light component of a customized light-dark box apparatus (dark compartment 0 lux; light compartment 100 lux; each chamber 30 cm long × 30 cm wide × 30 cm high) and allowed to freely explore for 10 min while video recorded from above. The time spent in the light component was analyzed as measures of anxiety-like behaviour.

#### 2.5.2. Elevated Plus Maze

Rats were placed in the center of the elevated plus maze apparatus (2 closed arms: each 50 cm long × 10 cm wide × 50 cm high, and 2 open arms: each 50 cm long × 10 cm wide × 1 cm high) and video recorded from above for 10 min. Customized tracking software (https://github.com/cdoolin/rat-apps, accessed on 1 September 2020) was used to quantify the percent time spent in the open arms and the total distance travelled. This test was used only once to avoid one-trial tolerance [24].

#### 2.5.3. Sucrose Preference Test

Rats were exposed to two water bottles in their home cage: one with a 2% sucrose solution and one with regular drinking water. The percentage of sucrose water consumed over 48 h was calculated as a measure of anhedonia. The location of the bottles was switched at 24 h to avoid side preference.

#### 2.5.4. Open Field

Rats were placed in the center of an open field arena (100 cm long × 80 cm wide × 30 cm high) and video recorded from above for 5 min. Offline video analysis was performed using customized tracking software (https://github.com/cdoolin/rat-apps, accessed on 1 September 2020) to quantify the total distance travelled.

#### 2.5.5. Cylinder

Rats were placed in an acrylic cylinder (21 cm diameter × 23 cm high) with mirrors located behind so that the rat could be observed from all sides using one camera. Each rat was video recorded for 3 min and offline analysis was used to quantify the number of left and right paw placements made on the side of the cylinder. Forepaw asymmetry was expressed as the percentage of ipsilesional paw placements.

#### 2.5.6. Von Frey Test

Rats were acclimatized to the testing chamber 5 min per rat the day before testing (IITC Life Science, Woodland Hills, CA, USA). Tactile sensitivity was assessed on both forepaws (when the animal was weight-bearing on its forepaws). A rigid probe connected to the automated Von Frey apparatus was applied in increasing pressure until the rat displayed a defined nociceptive response (paw retraction, licking) and the maximum pressure that elicited a withdrawal was recorded. This was repeated 3 times per paw, with a minimum of 3 min between measures. The average of the 3 measures per paw was used for analysis.

#### 2.5.7. Social Interaction

The test rat was placed in the open field apparatus with an unfamiliar, uninjured rat for 10 min while video recorded from above. The time spent in active interaction (sniffing, nipping, grooming, following, mounting, kicking, boxing, wrestling, jumping on, and crawling) was recorded as a measure of anxiety-like behaviour [25].

### 2.6. Fecal Collection and Transplantation

Fecal samples were collected as previously described [2]. During the dark cycle, rats were placed into individual sterile cages. Fecal pellets were immediately collected, placed into sterile eppendorf tubes and stored in a −80 °C freezer until further processing. For the fecal transplant solution, pellets were collected from uninjured FMT donors (pooled from all 10 rats as pooling samples from multiple donors has been shown to be more effective [26]) and immediately processed to make the transplant solution. The fresh fecal matter was diluted 1:10 in sterile PBS (10%), l-cysteine HCL (0.05%), glycerol (20%) and sterile water (60%) and passed through a 100 μm filter. The solution was frozen at −20 °C and thawed at room temperature for 12 h prior to use (the use of frozen fecal matter for oral FMT has proven to be effective [27]). The SCI + vehicle group received the filtered solution that did not contain fecal matter. Then, 2 h after SCI and for 2 consecutive days after, rats were gavaged with 500 μL of either FMT or vehicle solution.

### 2.7. 16S rRNA Sequencing

DNA was extracted as previously described [28]. Fecal microbial DNA was extracted with AquaStool solution (Multitarget Pharmaceuticals LLC, Colorado Springs, CO, USA) as per the manufacturer instructions. Briefly, 100 mg of rat fecal pellet was homogenized in the AquaStool solution with 0.1 mm beads at 0.6 m/s for 40 s. AquaRemove was added to remove potential PCR inhibitors per manufacturer’s instruction followed by ethanol/NaCl precipitation for further purification. DNA Samples were sent to Genome Quebec (McGill University, Montreal, QC, Canada) for Illumina Miseq sequencing. V3-V4 region of universal 16S rRNA primers with 341 forward primer: 5′-TCG TCG GCA GCG TCA GAT GTG TAT AAG AGA CAG CCT ACG GGN GGC WGC AG-3′ and 805 reverse primer: 5′-GTC TCG TGG GCT CGG AGA TGT GTA TAA GAG ACA GGA CTA CHV GGG TAT CTA ATC C-3′ were used.

Demultiplexed paired-end sequences were merged and performed quality control implementation (mean sequence quality score ≥ 30) and features table construction (amplicon sequences variants, ASVs) via DADA2 [29] plugin in QIIME2 (version 2019.10) [30]. An even sequence depth of 9452 reads per sample was used to conduct microbiome diversity and composition analyses. Taxonomy assignments from the phylum to genus levels were conducted by a pre-trained Naive Bayes classifier [30] (Silva 132 99% OTUs database) and the q2-feature-classifier function in QIIME2. Alpha-diversity of Shannon index and community balance of Pielou’s evenness index, and beta-diversity analysis (unweighted unifrac emperor distance) were conducted using the QIIME2.

### 2.8. Blood Collection

The area over the tarsal joint was shaved and the saphenous vein was punctured using a sterile needle. Blood was collected into a microvette CB300 capillary tube (Sarstedt Inc., Nümbrecht, Germany) and immediately centrifuged for 5 min at 3000 rpm. Plasma was then pipetted into sterile microcentrifuge tubes and stored at –80 °C freezer until further processing.

### 2.9. Cytokine Analysis

Frozen plasma samples were sent to Eve Technologies (Calgary, AB, Canada) and diluted 2-fold for the Rat Cytokine 27-Plex discovery assay. Cytokines and chemokines measured were: Eotaxin, EGF, Fractalkine, IFN-gamma, IL-1a, IL-1b, IL-2, IL-4, IL-5, IL-6, IL-10, IL-12(p70), IL-13, IL-17A, IL-18, IP-10, GRO/KC, TNF-alpha, G-CSF, GM-CSF, MCP-1, Leptin, LIX, MIP-1alpha, MIP-2, RANTES, and VEGF. GRO/KC values are not reported as they were out of range in our samples. For heatmap visualization, plasma analytes were expressed as a change from baseline (x_2_ − x_1_/x_1_).

### 2.10. Intestinal Permeability Assay

Once the uninjured FMT donor rats had completed all of their baseline testing and fecal collections, they were used to assess intestinal permeability. These rats were randomly divided into an SCI + vehicle group (*n* = 5) and an SCI + FMT group (*n* = 5) and received identical treatment as the original treatment groups (2 h after SCI and for 2 consecutive days after, rats were gavaged with 500 μL of either FMT or vehicle solution). The day before injury and again 7 days following SCI, rats were fasted for 4 h and then gavaged with 0.6 g/kg FITC dextran (4 kD, Sigma-Aldrich) diluted in sterile PBS. Blood was collected 4 h later via the saphenous vein and plasma was collected as described above. Plasma samples were diluted 1:10 with sterile PBS and transferred to an opaque-bottom 96-well plate. Samples were run in duplicates and a PBS blank and standard curve measurements were measured on the same plate. Fluorescence was determined at 530 nm with an excitation at 485 nm on a plate reader (SpectraMax, Molecular Devices, San Jose, CA, USA). Intestinal permeability was quantified as a fold change from baseline levels.

### 2.11. Perfusion and Tissue Cutting

At the end of rehabilitative training and all final behavioural assessments (78 days after SCI), rats were euthanized with sodium pentobarbital (240 mg/kg). Rats were transcardially perfused with saline containing 0.02 g heparin/L followed by 4% paraformaldehyde in 0.1 M phosphate-buffered saline (PBS) and 5% sucrose. Spinal cords were extracted and post-fixed in 4% paraformaldehyde 4 °C for 4 h and transferred to a 30% sucrose solution for 5 days. A 1 cm block around the lesion site was embedded in O.C.T. (Sakura Finetek, Torrance, CA, USA), mounted onto filter paper and frozen at −40 °C in 2-methylbutane. A NX70 cryostat (Fisher Scientific, Waltham, MA, USA) was used to section the cord at a thickness of 25 μm. Every second section was kept and staggered across eight slides and stored at −20 °C.

### 2.12. Lesion Analysis

Frozen slides were thawed for 1 h at 37 °C and washed in TBS (2 × 10 min). Slides were placed into 0.5% cresyl violet for 3 min, rinsed with filtered water and serially dehydrated in EtOH (2 min in 50%, 75%, and 99%). Slides were then placed in xylene (2 × 2 min) and coverslipped with Permount™. Images of the entire lesion extension were taken with an epifluorescence microscope (Leica DM6000B, camera Leica DFC350 FX, Wetzlar, Germany) at 5× magnification and analyzed using ImageJ (National Institute of Health, Bethesda, MD, USA). Lesion size was calculated as the percent of damaged tissue divided by the total area of the spinal cord cross section.

### 2.13. Analysis of IBA1 Staining

Sections were thawed at 37 °C for 1 h and rehydrated in PBS (2 × 10) minutes followed by PBS with 0.3% Triton™ X-100 (PBS-T) (1 × 10 min). Blocking buffer consisting of 5% normal donkey serum in PBS-T was applied 1 h at room temperature. Sections were incubated overnight at room temperature in rabbit-anti-IBA1 (1:500, Wako, Cape Charles, VA, USA) antibody (to visualize microglia/macrophages) with blocking buffer. The next day, sections were washed with PBS (3 × 10 min) and incubated with donkey-anti-rabbit AF488-conjugated (1:500, Life Technologies, Carlsbad, CA, USA) antibody in the blocking buffer solution for 2 h. Sections were then rinsed in PBS (2 × 10 min) and cover slipped with Fluoromount™. Images were captured with an epifluorescence microscope (Leica DM6000B, camera Leica DFC350 FX, Wetzlar, Germany) and analyzed using ImageJ (National Institute of Health, Bethesda, MD, USA). Then, 5× magnification images were taken to visualize the entire spinal cord cross section 0.25 cm rostral to the lesion, at the lesion epicenter, and 0.25 cm caudal to the lesion. The area of IBA+ immunoreactivity was divided by the total area of each individual spinal cord cross section and expressed as a percentage of IBA1+ area using thresholding.

### 2.14. Statistical Analysis

Statistical analyses were performed using GraphPad Prism 8 (San Diego, CA, USA) and an alpha value of 5% or less was considered significant. Normality was analyzed using the D’Agostino-Pearson omnibus test. Data at a single time point were analyzed using an unpaired parametric *t*-test for two groups and an ordinary one-way ANOVA for three groups (non-parametric tests were used for data that did not pass normality). Data with multiple time points were analyzed using an ordinary repeated measures two-way ANOVA followed by Sidak’s multiple comparison test.

## 3. Results

### 3.1. Fecal Microbiota Transplant from Anxious Donors

**Figure 2:**
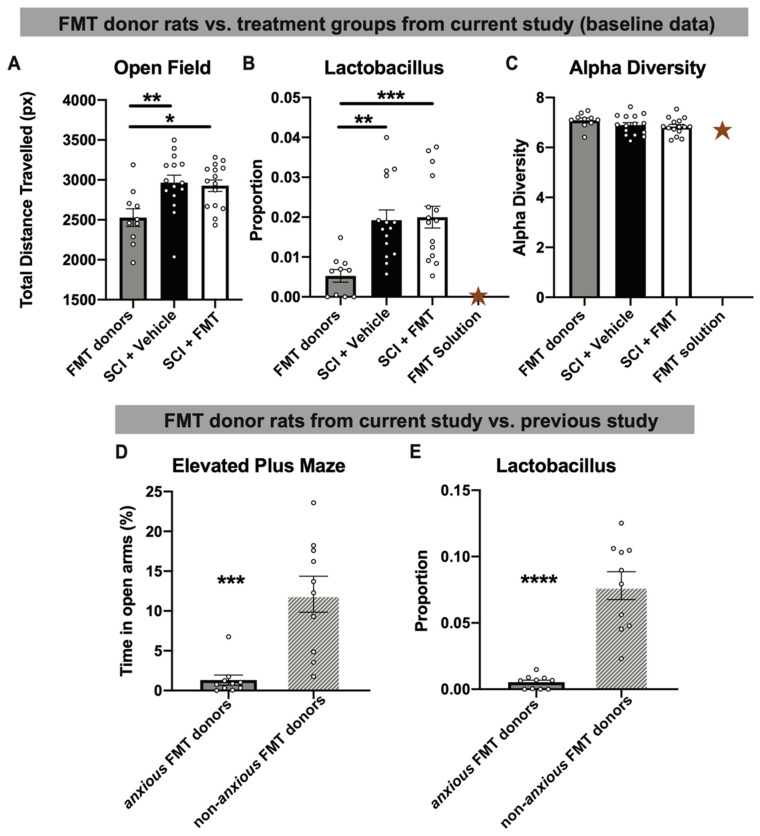
Uninjured FMT donor rats displayed altered baseline levels of anxiety-like behaviour and proportions of fecal *Lactobacillus*. (**A**) Fecal microbiota transplant (FMT) donors travelled significantly less distance in the open field compared to the SCI + vehicle and SCI + FMT treatment groups in the present experiment (measured at baseline prior to SCI). (**B**) Fecal matter from FMT donors had significantly decreased baseline proportions of *Lactobacillus*, which is also reflected in the decreased amount of *Lactobacillus* found in the FMT solution. (**C**) All groups had similar baseline levels of alpha diversity, including the FMT solution. (**D**) FMT donors in the current study displayed significantly increased anxiety-like behaviour in the elevated plus maze (indicated by the percent of time spent in the open arms) and (**E**) had significantly less fecal proportion of *Lactobacillus* relative to successful FMT donor rats from previous experiments. * *p* < 0.05, ** *p* < 0.01, *** *p* < 0.001, **** *p* < 0.0001. Gold star represents the FMT solution (A single value and therefore not included in statistical analysis). Error bars represent standard error mean.

Although the rats used in the present experiment are genetically identical siblings, there is a natural variability in their baseline levels of anxiety-like behaviour, which can be further influenced by environmental stressors. To determine how important optimal donor selection is, the two cages of rats who naturally displayed decreased baseline activity in the open field (as an indicator of anxiety-like behaviour [31,32]) were chosen as the FMT donors. Compared to SCI + vehicle and SCI + FMT groups at baseline (prior to SCI), FMT donors travelled significantly less distance in the open field (*p* = 0.0052) (Figure 2A). This altered behavioural phenotype was associated with significantly reduced levels of *Lactobacillus* in the FMT donor’s stool compared to the experimental groups (SCI + Vehicle and SCI + FMT) at baseline (*p* = 0.0006) (Figure 2B). Reflecting the lack of *Lactobacillus* in the donor stool, the FMT solution also contained a lack of *Lactobacillus* (Figure 2B). FMT donors displayed a similar alpha diversity (the bacterial variance within the samples) as the experimental groups, which was also reflected in the FMT solution (Figure 2C). Compared to previously successful FMT donors (which, when transferred to rats after SCI, prevented both SCI-induced dysbiosis and anxiety-like behaviour [2]), *anxious* FMT donors spent significantly less time in the open arms of the elevated plus maze, confirming their increased anxiety-like phenotype (*p* = 0.0002) (Figure 2D). The robustness of behaviour in the elevated plus maze of non-*anxious* uninjured rats throughout different cohorts of animals is shown in Supplementary Figure S1. Not only did *anxious* FMT donors spend significantly less time in the open arms compared to previous non-*anxious* donors, they also displayed significant increased anxiety-like behaviour compared to two separate groups (from different experiments) of uninjured animals run in the elevated plus maze (*p* = 0.0006). *Anxious* FMT donors also displayed significantly lower proportions of *Lactobacillus* compared to the non-*anxious* FMT donors described in our previous study (*p* < 0.0001; [2]) (Figure 2E). These data suggest that, although the FMT donors were uninjured and had a diverse microbiota composition, they had an increased anxiety-like phenotype and reduced proportion of the genus *Lactobacillus*, a commonly prescribed probiotic [33,34,35].

### 3.2. FMT from Anxious Rats Did Not Prevent Dysbiosis after SCI

**Figure 3:**
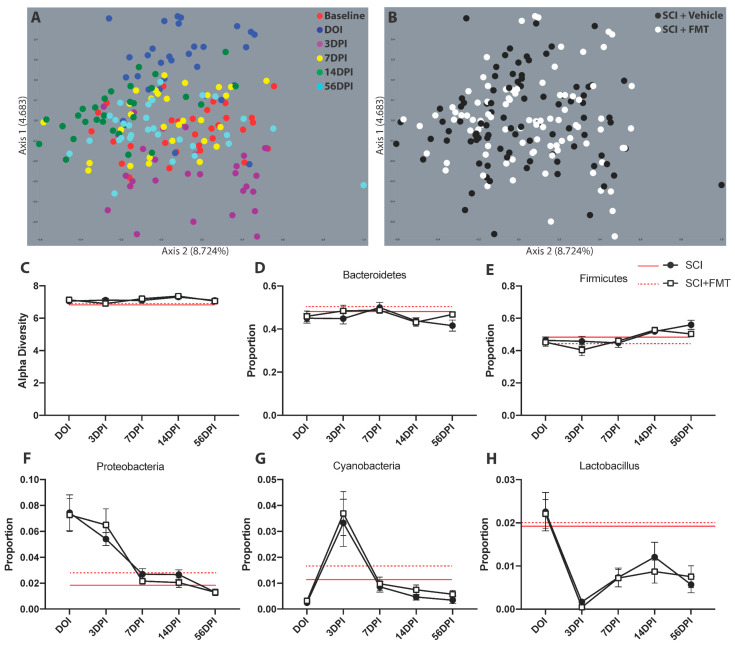
FMT from *anxious* donors did not prevent gut dysbiosis following SCI. (**A**) PCoA plot of beta diversity shows the diversity between fecal samples over time on the day of injury (DOI), 3-, 7-, 14- and 56-days post-injury (DPI). (**B**) The same PCoA plot is shown with the colors representing the groups instead of timepoints. Axis 1 and 2 explain 4.683% and 8.724% of the variance between samples, respectively. (**C**) There was no effect of injury or treatment on the alpha diversity. The four most abundant operational taxonomic units at the phylum level also show no differences between experimental groups in the proportion of (**D**) Bacteroidetes, (**E**) Firmicutes, (**F**) Proteobacteria and (**G**) Cyanobacteria. (**H**) The proportion of the genus *Lactobacillus* was reduced after SCI but not affected by FMT. Red lines represent baseline values. Error bars represent standard error mean.

Fecal samples were collected prior to injury, on the day of injury, then 3, 7, 14 and 56 days after SCI for 16S rRNA sequencing. The differences in microbial abundance between the fecal samples was visualized using beta diversity plots. On the day of injury, 3- and 14-days post-SCI there was a deviation in the samples away from baseline values, confirming our previous results that a cervical SCI induces acute dysbiosis. At 7- and 56-days post-SCI, the samples clustered closely with baseline values (Figure 3A). When looking at the beta diversity of the two treatment groups across all time points, there was no difference between FMT or vehicle treated groups (Figure 3B). Although SCI resulted in acute dysbiosis visualized in the beta diversity plots, there was no significant effect of injury or FMT on the alpha diversity (which does not necessarily correlate with changes of individual bacteria; Figure 3C). Next, we looked at the four most abundant bacteria at the Phylum level: Bacteroidetes, Firmicutes, Cyanobacteria and Proteobacteria. There was no effect of SCI or FMT in the proportion of Bacteroidetes or Firmicutes (Figure 3D,E). The proportion of Proteobacteria was increased on the day of injury and 3 days post injury (Figure 3F) and the proportion of Cyanobacteria was increased 3 days post-SCI (*p* < 0.0001 for both) (Figure 3G), however there were no significant effects of FMT treatment. The proportion of the genus *Lactobacillus*, a common bacteria present in probiotics [34], was reduced chronically after SCI in both FMT treated and untreated groups (*p* < 0.0001) (Figure 3H). There was no significant difference between groups in any bacteria at the genus level (Supplementary Figures S2 and S3). These results indicate that the FMT from *anxious* donor rats was not successful in preventing SCI-induced dysbiosis.

### 3.3. FMT from Anxious Rats Did Not Affect Functional Recovery from SCI

**Figure 4:**
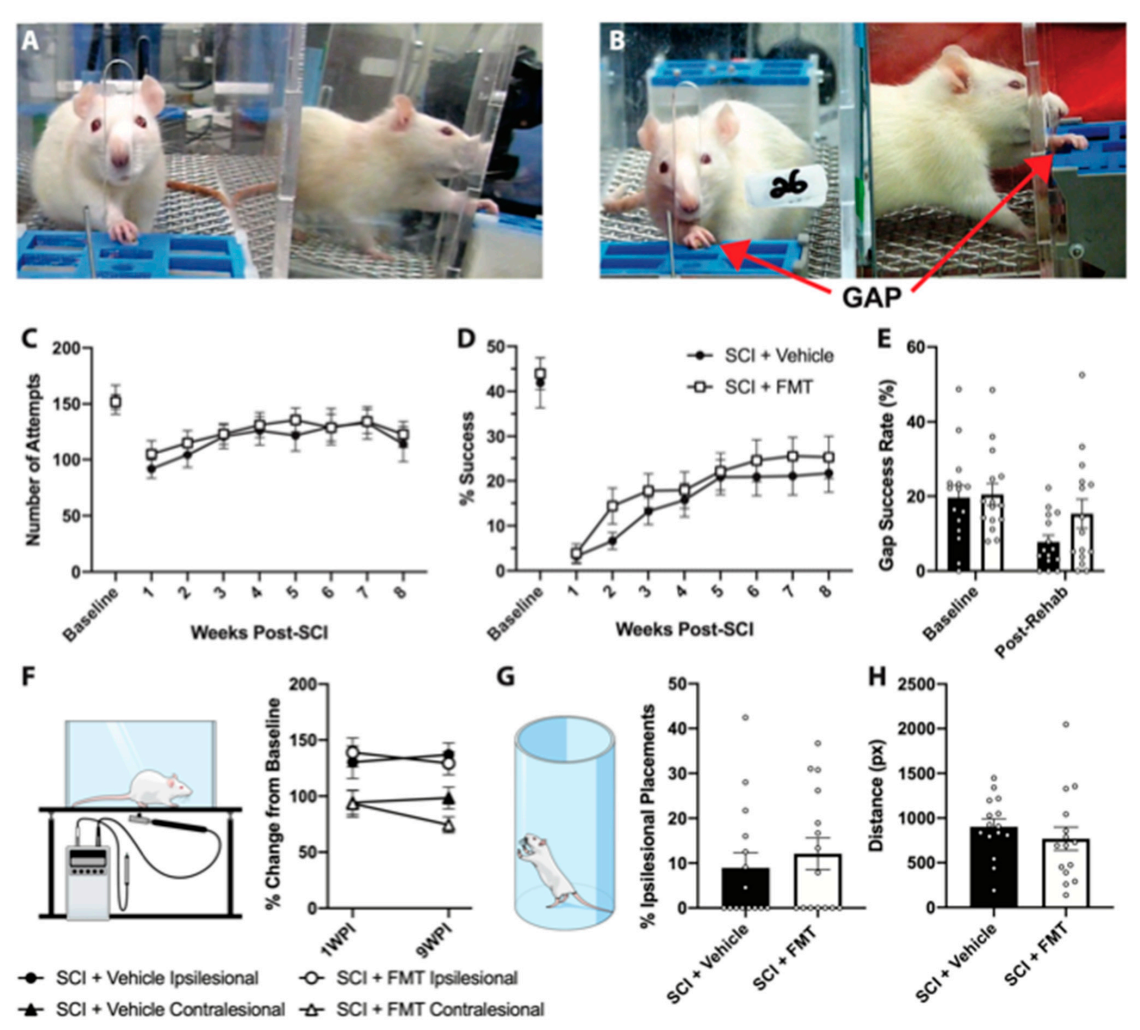
FMT from *anxious* rats did not significantly affect motor recovery following spinal cord injury. (**A**) Image of a rat in the regular single pellet grasping apparatus, reaching through a narrow opening for a food pellet. (**B**) Image of a rat reaching in the single pellet grasping apparatus that has been modified to include a gap between the pellet and the opening of the chamber (to eliminate compensatory pellet scooping behaviour). (**C**) There was no difference between FMT and vehicle groups in the number of attempts or (**D**) the success rate in rehabilitative training. (**E**) The success rate in the modified gap task was measured once at baseline and again at the end of the rehabilitation period. There were no significant differences between FMT and vehicle treated groups in the von frey test (quantified as the force required to elicit a withdrawal response, expressed as a percentage of baseline values) (**F**) the cylinder test (**G**) or the distance travelled in the open field (**H**). Error bars represent standard error mean.

**Figure 5:**
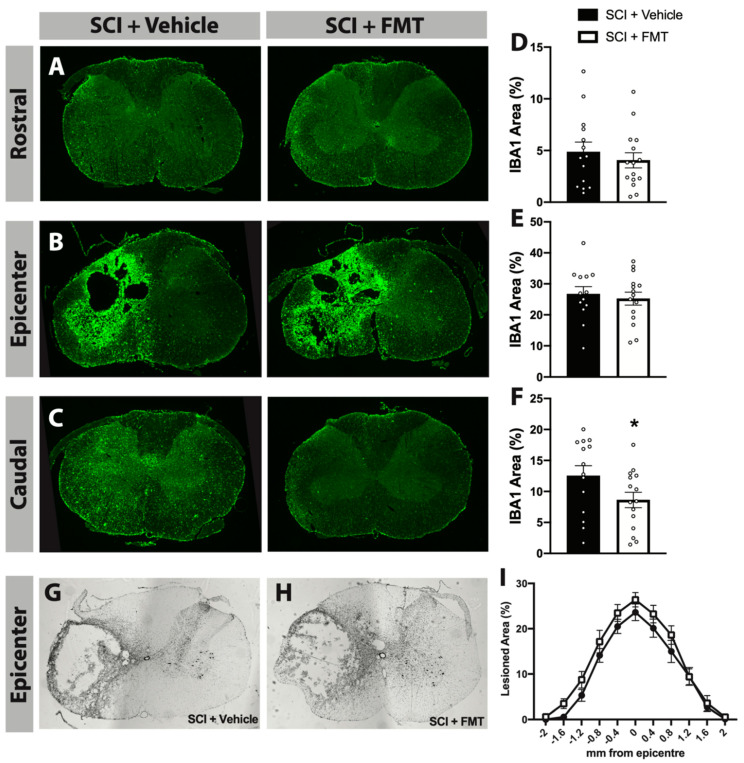
FMT from *anxious* donors reduced the area of IBA1+ cells caudal to the injury. Representative images of IBA1+ positive cells in the cervical spinal cord immediately rostral to the injury (**A**), at the injury epicenter (**B**) and immediately caudal to the injury (**C**). The percentage of IBA1+ area per spinal cord cross section rostral to, at and caudal to the lesion is quantified in (**D**–**F**), respectively. Immediately caudal to the injury, SCI + FMT rats displayed significantly reduced IBA1+ area compared to vehicle controls. Representative cross sections of the maximum injury site for SCI + Vehicle and SCI + FMT groups are shown in (**G**,**H**), respectively. (**I**) Quantification of the rostral (negative measurements) to caudal (positive measurements) extension of the lesion area was expressed as a percentage of lesioned tissue. * *p* < 0.05. Error bars represent standard error mean.

10 days following SCI, rats began 7 weeks of rehabilitative therapy in the SPG task which targeted their impaired forepaw (Figure 4A). There was no difference between FMT or vehicle treated rats in the number of attempts made to reach for the pellet, indicating that the FMT did not influence participation in rehabilitation (Figure 4C). There was a significant decrease in success rate following SCI, which gradually improved for both vehicle and FMT groups throughout the rehabilitation period (Figure 4D). To prevent compensatory pellet-scooping strategies, rats were tested in a modified task where a gap was introduced between the pellet and the training chamber (Figure 4B). There was a trend for FMT rats to perform better in the gap test at the end of the rehabilitation period, however this did not reach statistical significance (*p* = 0.089) (Figure 4E). FMT treatment did not alter mechanical sensitivity, however both groups experienced reduced sensitivity of the ipsilesional forepaw at 7 and 63 days post injury (Figure 4F). At the end of the rehabilitative training period, rats were tested in the cylinder task to measure forepaw asymmetry and in the open field to assess locomotor activity; there were no differences between groups in either of these tests (Figure 4G,H). Although there was no significant treatment effect in the efficacy of rehabilitative training or motor recovery following SCI, treatment with FMT from *anxious* donors resulted in a chronic (77 days post injury) decrease in the percentage area of IBA+ immunoreactivity caudal to (*p* = 0.046), but not rostral to or at, the lesion site compared to vehicle controls (Figure 5A–F). This decreased area of IBA+ cells was not due to differences in injury size, as the lesion extension and area were similar between groups (Figure 5G–I).

### 3.4. FMT from Anxious Donors Increased Anxiety-Like Behaviour

**Figure 6:**
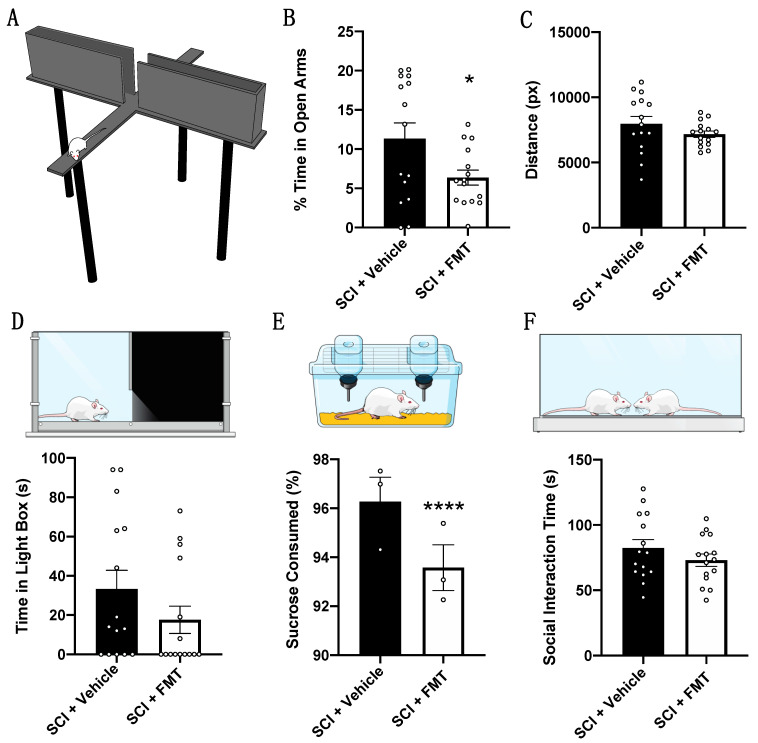
FMT from *anxious* donors resulted in a chronic increase in anxiety-like behaviour after SCI. At the end of rehabilitative training, rats were tested for anxiety-like and depressive-like behaviours. (**A**) Schematic of a rat in the open arm of the elevated plus maze. (**B**) SCI + FMT rats spent significantly less time in the open arms compared to untreated rats. (**C**) Both groups of rats travelled a similar amount of distance in the elevated plus maze. (**D**) SCI + FMT rats spent less time in the light-component of the light-dark box and (**E**) drank less sucrose water than untreated rats (each data point represents a cage containing 5 rats, each of which were considered for statistical analyses). (**F**) Both fecal transplant treated and untreated rats spent a similar amount of time interacting in the social interaction test. * *p* < 0.05, **** *p* < 0.0001. Error bars represent standard error mean.

At the end of rehabilitative training, rats were tested for depressive- and anxiety-like behaviours. Rats that received an FMT from *anxious* donors spent significantly less time in the open arms of the elevated plus maze (*p* = 0.0341), although both groups travelled a similar total distance (Figure 6A–C). The magnitude of differences between groups in the open arms is less than that observed between *anxious* and non-*anxious* FMT donors (Figure 2D). Furthermore, SCI + vehicle rats displayed less anxiety-like behaviour than untreated SCI control rats in our previous study [2], which may be due to the daily rehabilitative training received in the present study. There was also a trend for the SCI + FMT group to spend less time in the light component of the light-dark box (Figure 6D) and they drank significantly less sucrose solution (*p* < 0.0001) (Figure 6E) compared to vehicle controls. Both FMT and vehicle groups spent a similar amount of time interacting in the social interaction test (Figure 6F).

### 3.5. Temporal Profile of Plasma Analytes Following Spinal Cord Injury

**Figure 7:**
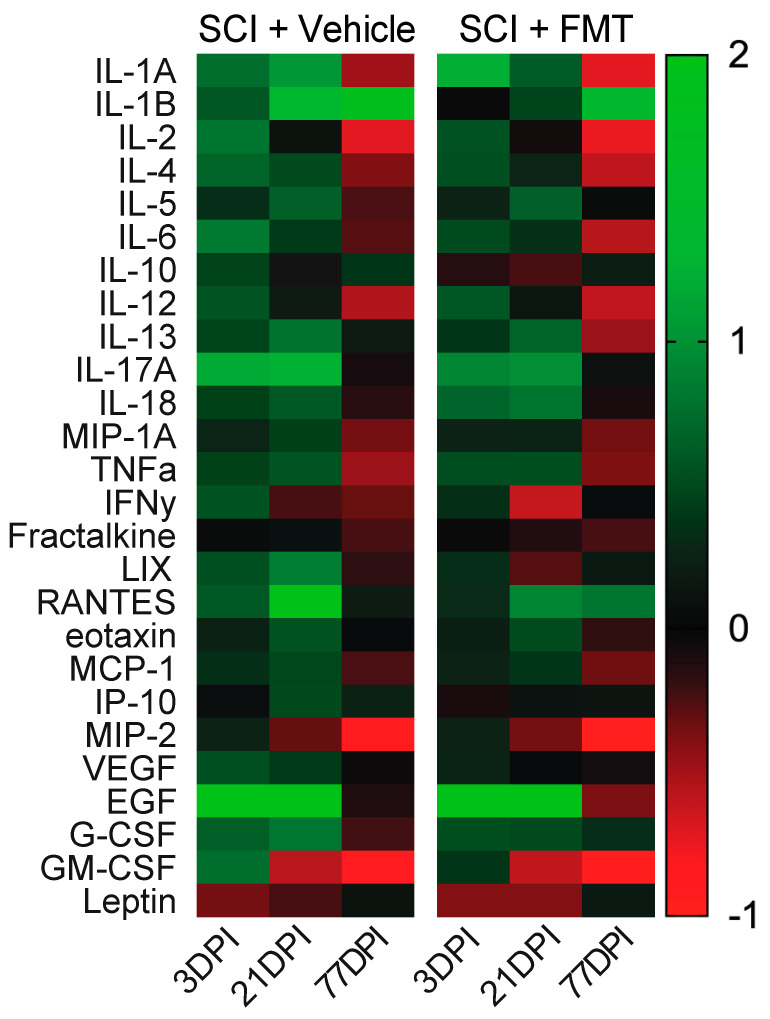
Heatmap of plasma markers over time following SCI. Plasma markers (cytokines, chemokines, growth factors, glycoproteins and hormones) were expressed as a change from baseline values and plotted over time (positive numbers represent an increase from baseline values and negative numbers represent a decrease from baseline values). Values above 2 were set at 2 for visualization purposes (RANTES and EGF were affected).

**Figure 8:**
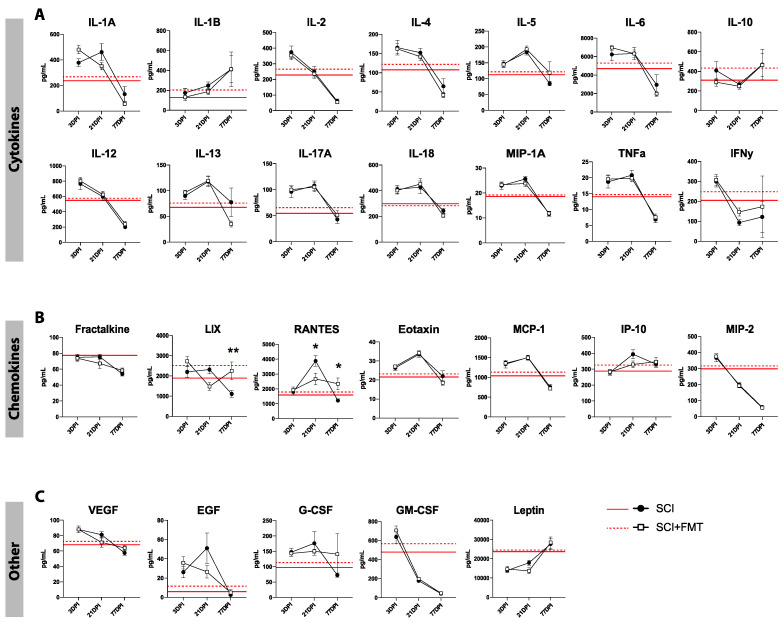
SCI induced time-dependent changes in plasma cytokines and chemokines. (**A**) Temporal profile of plasma cytokines 3 days post injury (3DPI), 21 days post injury (21DPI) and days post injury (77DPI) for SCI + Vehicle and SCI + FMT groups. (**B**) Temporal profile of plasma chemokines show that SCI + FMT rats have significantly increased levels of LIX and RANTES at 77 days compared to vehicle controls. (**C**) Profile of other plasma markers (growth factors, glycoproteins and hormones) over time after injury. Red lines represent baseline values. * *p* < 0.05. ** *p* < 0.01. Error bars represent standard error mean.

To determine the effect of both SCI and the FMT on acute and chronic systemic inflammation, plasma analytes were measured before SCI, then 3, 21 and 77 days after injury. There was an overall trend of increased levels of all plasma analytes at 3- and 21-days post SCI, and a drastic downregulation by 77 days in both experimental groups (Figure 7). Looking at the concentrations of each plasma analyte over time, rats which received the FMT displayed significantly increased concentration of LIX at 77 days (*p* = 0.009), reduced levels of RANTES at 21 days (*p* = 0.012) and higher levels of RANTES by 77 days post injury (*p* = 0.023) (Figure 8B). There was no significant treatment effect in any of the other chemokines, cytokines or other analytes measured (growth factors, glycoproteins and the hormone leptin) (Figure 8A,C).

### 3.6. FMT from Anxious Donors Increased Intestinal Permeability

**Figure 9:**
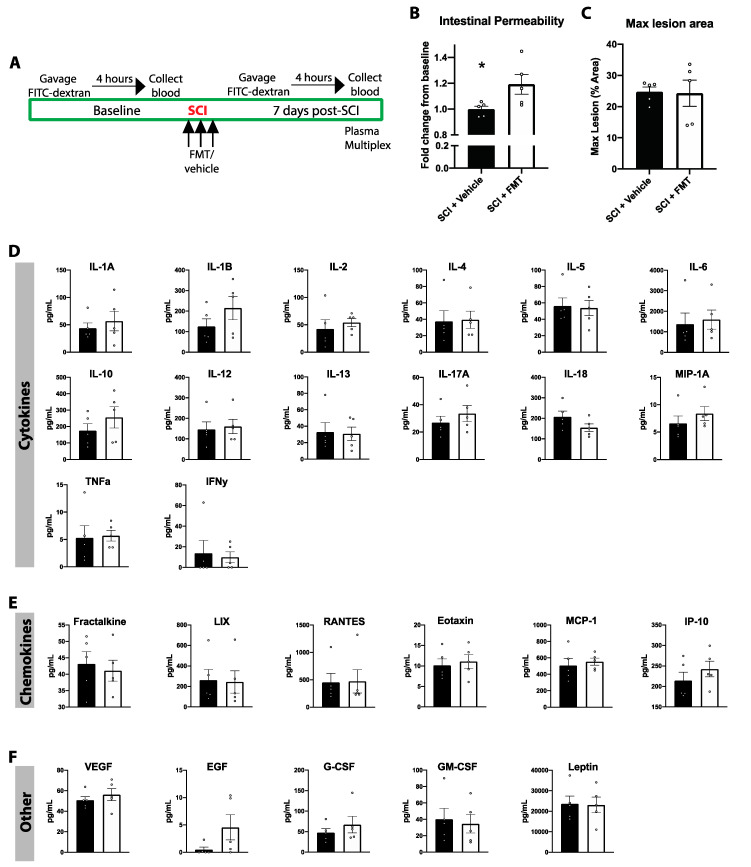
FMT from *anxious* donors increased intestinal permeability. (**A**) The FITC-dextran test for intestinal permeability was performed at baseline prior to spinal cord injury and again 7 days after injury. (**B**) SCI + FMT rats displayed significantly increased intestinal permeability relative to vehicle controls. (**C**) There were no differences between groups in the maximum lesion size. 7 days following injury, plasma was extracted and analyzed for levels of various cytokines (**D**), chemokines (**E**) and other growth factors, glycoproteins and hormones (**F**). * *p* < 0.05. Error bars represent standard error mean.

Increased intestinal barrier permeability has previously been shown in mice 7 days following a thoracic SCI, which can allow bacterial and other matter to translocate across the impaired epithelial tight junctions [4,36]. To test whether a cervical contusion SCI in rats also triggers an increase in intestinal permeability, rats were gavaged with FITC-dextran and the concentration of FITC was measured in blood 4 h later (Figure 9A). This test was performed before SCI and again 7 days later and expressed as a fold change from baseline to account for individual differences. SCI alone did not alter intestinal permeability, however FMT from *anxious* donors increased intestinal permeability by nearly 20% compared to baseline (SCI + Vehicle vs. SCI + FMT *p* = 0.043) (Figure 9B). This increased intestinal permeability was not due to differences in lesion size (Figure 9C). To determine whether differences in intestinal permeability between groups was associated with changes in systemic inflammation at the same time, plasma cytokines/chemokines were analyzed in these rats 7 days post injury. There was no difference between FMT or vehicle controls in plasma concentrations of cytokines, chemokines, or other growth factors, glycoproteins and hormones (Figure 9D–F).

## 4. Discussion

The use of healthy human stool to treat diseases has been documented in Chinese medicine for over 1700 years [37]. However, the first report of FMT treatment in modern Western medicine was not until 1958 [38], and it was not until 2013 that FMT was included in the treatment guidelines for recurrent *Clostridium difficile* infections [39]. The popularity of FMT as a treatment is increasing rapidly for various other diseases, such as: irritable bowel disease, irritable bowel syndrome, obesity, autism, Parkinson’s disease, multiple sclerosis, metabolic syndrome, stroke and SCI [2,8,14,40,41,42,43,44,45,46,47,48]. Aside from excluding donors with known fecal matter pathogens, the selection of FMT donor does not appear to influence the success of treatment for *Clostridium difficile* infection [9,21]. However, the same is not necessarily true for other disorders, especially those with more complicated microbiota-disease interactions such as SCI. Donor selection criteria beyond the exclusion of known pathogens is therefore a crucial area of research that is still in its infancy [15,19].

Previously we have shown that FMT from uninjured, non-*anxious* rats prevented both acute dysbiosis and the development of anxiety-like behaviour following SCI [2]. Contrary to our hypothesis, here we show that optimal donor selection is essential for successful (i.e., prevents SCI-induced dysbiosis) FMT treatment following SCI. Critically, the FMT donor rats in the present study were uninjured, free of pathogens and are genetically compatible to the recipients and would likely have passed screening criteria used clinically for FMT donors. In FMT trials, potential donors undergo a preliminary interview to rule out potential risk factors such as drug use and medical history [15,19,49,50,51]. Individuals who pass the preliminary interview then undergo blood and stool testing to exclude the risk for transferring infectious diseases [15,19,50,51]. Although a history of psychiatric conditions is a risk factor for potential FMT donors [52], it is often not considered for donor screening [15,19,49,50,51]. This is particularly relevant for studies on the efficacy of FMT for depression and anxiety. While there are relatively few human studies on FMT for treating psychiatric disorders, the existing results show short-term success but inconsistent long-term improvement [53,54,55,56,57]. The results of the present study in rats suggest that even minor behavioural abnormalities can impact the success of FMT and may help explain the inconsistent long-term results of FMT treatment for psychiatric disorders. Indeed, multiple animal studies show that the behaviour of the FMT donor can be transferred to the recipient [58,59,60,61,62].

In the present study, the FMT donors had increased baseline levels of anxiety-like behaviour which was associated with a significant reduction in the proportion of *Lactobacillus* in their stool. Although a causal relationship between gut bacteria and the development of mental health disorders has not been shown, many studies have found a strong association between the two. For example, humans diagnosed with major depressive disorder have reduced levels of *Lactobacillus* compared to controls [63]. Furthermore, *Lactobacillus* is one of the most frequently used probiotic bacteria and has been shown to improve anxiety and depression in multiple preclinical studies [64,65,66] and clinical trials [67,68,69]. In a recent double-blind, randomized, placebo controlled study, treatment with the probiotic *Lactobacillus* was shown to significantly reduce kynurenine concentrations in patients with major depressive disorder [70]. The kynurenine pathway can be activated by inflammation and is thought to play a significant role in the pathogenesis of depression [71,72]. Reducing kynurenine concentrations by blocking indoleamine 2,3-dioxygenase (the rate-limiting enzyme in the kynurenine pathway of tryptophan metabolism [73]) has also been shown to block lipopolysaccharide (LPS) induced depressive-like behaviour in rodents [74]. The kynurenine pathway may therefore be an important player in the microbiota-immune-brain axis involved in the pathogenesis of depression and anxiety following SCI. The lack of *Lactobacillus* present in the FMT donor stool may indicate alterations in the kynurenine pathway and be, at least, partly responsible for the unsuccessful FMT. However, there were no significant differences between FMT and vehicle groups in the proportion of *Lactobacillus* following SCI at the time points measured. More detailed sequencing may be required to detect differences at the species level, as there are over 260 metabolically unique *Lactobacillus* strains and only some species are used in probiotics [34,75]. Nonetheless, sequencing at the Phylum level indicated a global acute shift in the microbiota composition on the day of injury and 3 days post-SCI which returned to baseline by 35 days, similar to previously reported [2]. However, in the present study, using FMT from *anxious* donors with low levels of *Lactobacillus* was unsuccessful in preventing SCI-induced dysbiosis.

Although the FMT from *anxious* donors used in the present study did not improve SCI-induced dysbiosis, there were some long-term effects on inflammation and anxiety-like behaviour. There is a strong link between increased inflammation and the development of mental health disorders [76,77]. In rodent models of SCI, increased local (brain and spinal cord tissue) and systemic inflammation have been associated with the development of anxiety and depressive-like behaviours [78,79]. Here, rats that received the FMT from *anxious* donors displayed increased anxiety-like behaviour, which may suggest an increased inflammatory phenotype. In support of this, FMT from *anxious* donors resulted in increased intestinal permeability measured 7 days after SCI. As a potential confound to this test, the intestinal permeability assay was run in rats with increased baseline levels of anxiety-like behaviour. Since stress itself can alter intestinal permeability, this may explain why we did not observe a change in intestinal permeability following SCI in control rats. Nonetheless, FMT from *anxious* donors increased the gut permeability of FMT recipient rats, which can allowed bacterial matter such as LPS to translocate across the impaired epithelial tight junctions [4,80]. Once in circulation, LPS triggers a strong immune response that can reach the central nervous system and last for months after exposure [81,82]. Recently, we showed that systemic injection of LPS following cervical SCI in rats induced a chronic increase in anxiety-like behaviour in the elevated plus maze [83]. Furthermore, rats that received LPS displayed enhanced recovery in rehabilitative training and a paradoxical reduction in microglial and astrocyte density around the lesion site [83]. Similar findings were observed in the present study; although not statistically significant, FMT treated rats displayed improved motor recovery in the modified gap test. Additionally, in line with our previous research, we found that the increased anxiety-like behaviour did not interfere with willingness of the rats to participate in rehabilitative training (as evidenced by their similar attempt rates across groups) [83]. Furthermore, rats that received the FMT also displayed significantly reduced area of IBA1+ cells caudal to the lesion site. These parallels between LPS and treatment with FMT from *anxious* donors provide credence to the hypothesis that the long-term side effects of FMT from *anxious* donors are a result of endotoxin translocation from a permeable intestinal barrier [36]. Although we did not measure systemic LPS, the chemokines LIX and RANTES (both of which are upregulated by LPS) were significantly increased in FMT treated rats 77 days after injury. RANTES mediates the trafficking of immune cells such as T cells, monocytes, natural killer cells and mast cells, whereas LIX is best known for recruiting neutrophils [84,85]; both chemokines are associated with a variety of inflammatory disorders. Therefore, increased concentrations of LIX and RANTES may suggest a chronic systemic inflammatory state compared to vehicle controls, however, further evidence would be required to substantiate this claim. In both groups, we observed a significant increase in both pro-inflammatory and anti-inflammatory cytokines and chemokines at 3 and 21 days after SCI. This is likely due to the acute systemic inflammatory response initiated following trauma to the spinal cord [86,87]. By 77 days, both FMT and vehicle groups displayed a drastic downregulation in the majority of inflammatory cytokines, which may reflect a symptom of SCI-induced immune depression [88]. This immune depression is hypothesized to be triggered by sympathetic dysregulation associated with upper thoracic and cervical SCIs and generally takes time to develop following injury [89,90].

## 5. Conclusions

In conclusion, these results highlight the importance of optimal donor selection for successful FMT treatment following SCI. Although the FMT donors were otherwise healthy and pathogen free, they displayed naturally increased anxiety-like behaviour and reduced proportions of *Lactobacillus*. FMT from these *anxious* donors did not prevent SCI-induced dysbiosis and had some negative side effects including increased intestinal permeability, increased anxiety-like behaviour, and minor yet chronic alterations in both local and systemic inflammation. Future work should investigate whether specific bacteria (such as *Lactobacillus)* are required for successful FMT as well as the optimal timing and dosage of treatment. While recipient safety must prevail above all, vigilant donor selection beyond the exclusion of known pathogens is essential to improve the success of FMT as shown here in the context of SCI.

## Data Availability

The data presented in this study are openly available from at https://scicrunch.org/odc-sci, accessed on 1 September 2020 (DOI: 10.34945/F5XW2P).

## Supplementary Materials

The following are available online at https://www.mdpi.com/2079-7737/10/4/254/s1, Figure S1: Percent time in the open arms of the elevated plus maze compared between anxious FMT donors, non-anxious FMT donors, and two other cohorts of rats from previous experiments. Figures S2 and S3: Microbiota changes at the genus level.
