# Supplementary figures and images for: What Makes a Successful Donor? Fecal Transplant from Anxious-Like Rats Does Not Prevent Spinal Cord Injury-Induced Dysbiosis
Source: Biology (Basel). 2021 Mar 24;10(4):254. doi: 10.3390/biology10040254 (PMC8063845; doi:10.3390/biology10040254)

● SCI  
□ SCI+FMT

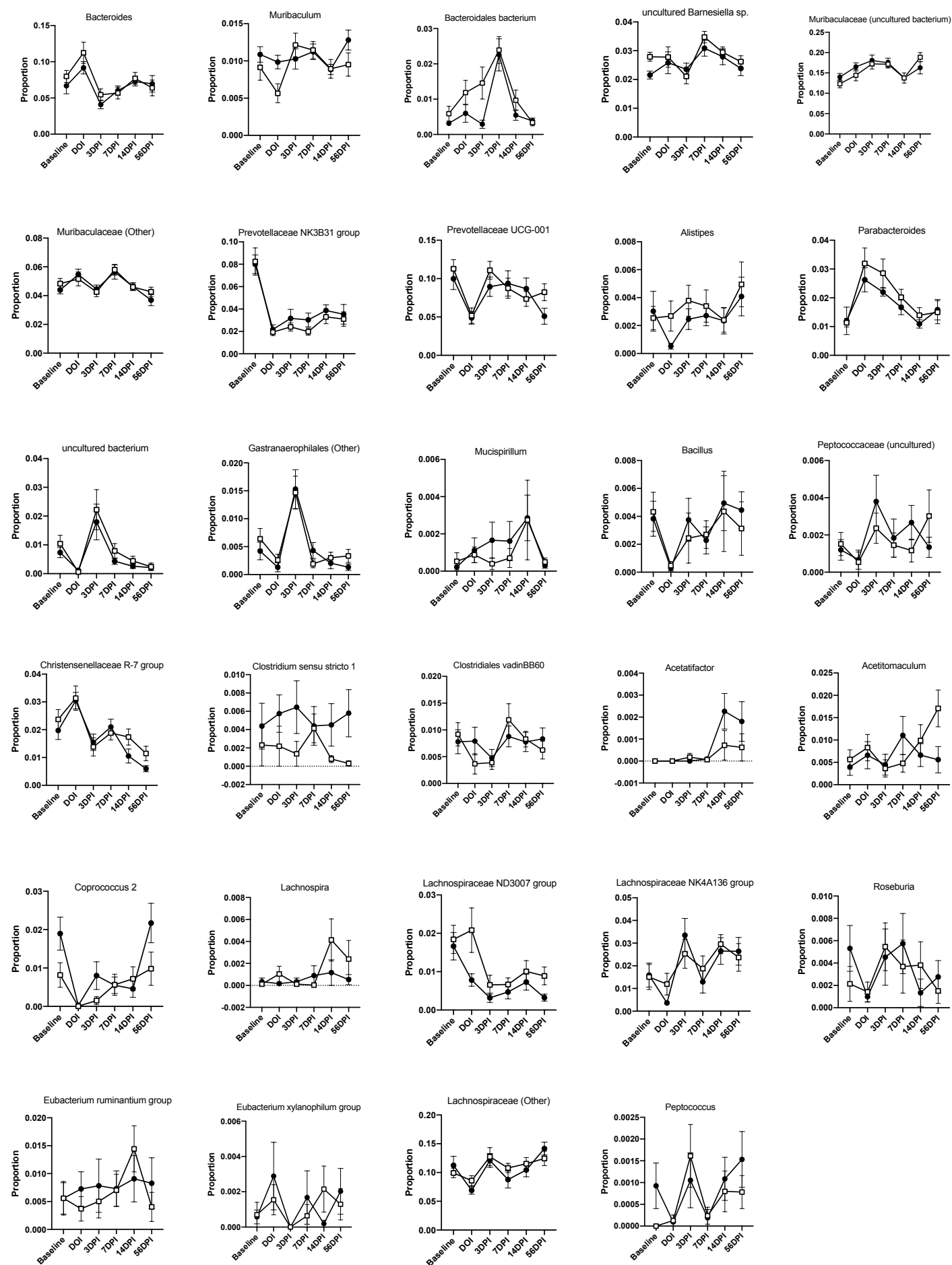

Supplement: Supplementary file 1 [file biology-10-00254-s001.zip › biology-1127094-supplementary (1)/S2.pdf]

● SCI  
 □ SCI+FMT

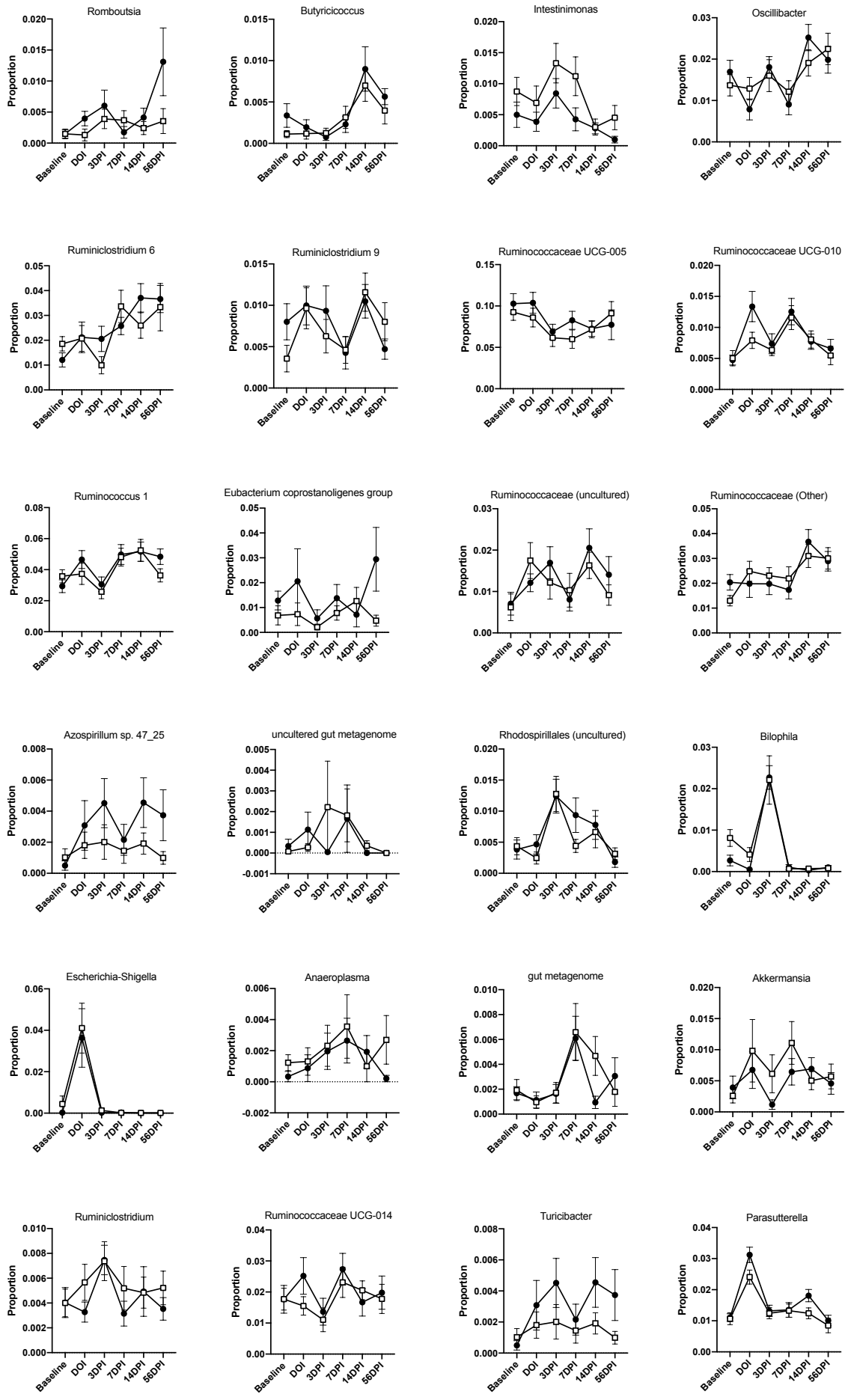

Supplement: Supplementary file 1 [file biology-10-00254-s001.zip › biology-1127094-supplementary (1)/S3.pdf]

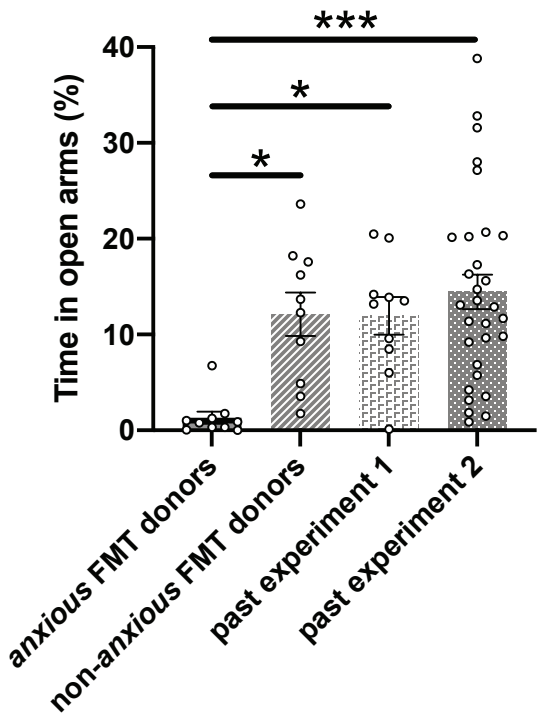

Supplement: Supplementary file 1 [file biology-10-00254-s001.zip › biology-1127094-supplementary (1)/S1.pdf]
